# Shaping the Trans-Scale Properties of Schizophrenia *via* Cerebral Alterations on Magnetic Resonance Imaging and Single-Nucleotide Polymorphisms of Coding and Non-Coding Regions

**DOI:** 10.3389/fnhum.2021.720239

**Published:** 2021-09-09

**Authors:** Shu-Wan Zhao, Xian Xu, Xian-Yang Wang, Tian-Cai Yan, Yang Cao, Qing-Hong Yan, Kun Chen, Yin-Chuan Jin, Ya-Hong Zhang, Hong Yin, Long-Biao Cui

**Affiliations:** ^1^Department of Clinical Psychology, School of Medical Psychology, Fourth Military Medical University, Xi'an, China; ^2^Department of Radiology, Xijing Hospital, Fourth Military Medical University, Xi'an, China; ^3^Department of Radiology, The Second Medical Center, Chinese PLA General Hospital, Beijing, China; ^4^Department of Psychiatry, Xijing Hospital, Fourth Military Medical University, Xi'an, China; ^5^Department of Anatomy and K. K. Leung Brain Research Centre, Fourth Military Medical University, Xi'an, China

**Keywords:** schizophrenia, magnetic resonance imaging, gene, trans-scale, single-nucleotide polymorphism

## Abstract

Schizophrenia is a complex mental illness with genetic heterogeneity, which is often accompanied by alterations in brain structure and function. The neurobiological mechanism of schizophrenia associated with heredity remains unknown. Recently, the development of trans-scale and multi-omics methods that integrate gene and imaging information sheds new light on the nature of schizophrenia. In this article, we summarized the results of brain structural and functional changes related to the specific single-nucleotide polymorphisms (SNPs) in the past decade, and the SNPs were divided into non-coding regions and coding regions, respectively. It is hoped that the relationship between SNPs and cerebral alterations can be displayed more clearly and intuitively, so as to provide fresh approaches for the discovery of potential biomarkers and the development of clinical accurate individualized treatment decision-making.

## Introduction

Schizophrenia is a common and complex multidimensional disease with high heredity, and genetic factors play an important role in its pathophysiological mechanism (McCutcheon et al., 2020). Understanding the genetic basis of schizophrenia is of great help to explore its pathogenesis.

In recent years, the application of trans-scale and multi-omics methods that combine gene and imaging in schizophrenia has promoted the elucidation of gene-related pathogenesis and the exploration of potential biomarkers (Reddaway et al., 2018). Trans-scale analysis is a research strategy for the joint analysis of information between different scales (van den Heuvel et al., 2019). From the macroscopic level, the human brain can be viewed as a complex network system made up of structural and functional connections between brain regions. And, from the microscopic perspective, the neurons containing dendrites and axons form a complex system of wiring which makes up the structural basis of our brain. Microscale level information, such as genetic, molecular, and cellular, can provide sufficient evidence for the construction of brain phenotypes and mechanisms of brain injury at the macroscale level (van den Heuvel et al., 2019). Multi-omics analysis is a research strategy of integrative analysis that integrates information between different disciplines. Many disciplines, including genomics, epigenomics, transcriptomics, proteomics, metabolomics, gut microbiomics, and connectomics, have made significant contributions to the study of the pathogenesis of schizophrenia (Guan et al., 2021). The multi-omics analysis strategy can provide a more comprehensive perspective on the exploration of schizophrenia pathogenesis.

With the evolvement of functional magnetic resonance imaging (fMRI), structural magnetic resonance imaging (sMRI), diffusion-weighted imaging (DWI), and other sequences, alterations of brain structure and function can be displayed more accurately on MRI. Brain microstructures, such as white matter myelination, can be measured indirectly in a non-invasive manner using magnetization transfer imaging (MTI) technique (Whitaker et al., 2016). MRI plays an increasingly important role in the study of brain phenotypes and differential diagnosis of mental disorders. Previous studies have confirmed that there are some alterations in brain structure and function in patients with schizophrenia (Brown and Thompson, 2010), which may be related to clinical symptoms of schizophrenia (Cui et al., 2017; Liu et al., 2019). Another study has found out that quantitative and specific functional connectivity (FC) biomarkers could be an effective radiomics features for individualized diagnosis for schizophrenia (Cui et al., 2018). The appearance of brain phenotypes on MRI may provide clues to the differential diagnosis of schizophrenia and bipolar disorder, which overlap in risk genes and clinical symptoms. While brain disturbances in patients with bipolar disorder are primarily located in the fronto-limbic subsystems, schizophrenia is characterized by disorders of the small world and rich club and the effects still present in the unaffected offspring (Perry et al., 2019). Therefore, MRI-based imaging study has gradually become the most common method to study mental disorders at macroscale, providing connectomics and radiomics approaches.

Sinlge-nucletode polymorphisms (SNPs) are the most common genetic variation at the genome level, which are caused by the change of a single base pair in the DNA sequence. SNPs are found randomly in the coding region or the non-coding region of the gene and produce corresponding effects by affecting gene expression, mRNA processing, and protein translation (Bush and Moore, 2012; Gurung and Prata, 2015; Roy et al., 2020). According to the research, SNPs account for a large part of the genetic variation associated with schizophrenia (Pardinas et al., 2018). Although recent genome-wide association studies (GWASs) have identified many SNPs loci associated with schizophrenia (Schizophrenia Working Group of the Psychiatric Genomics C., 2014), providing many possible genetic variation resources with biological functions for analyzing the pathogenesis of schizophrenia, the functions behind these mutations still need to be verified. Changes in genes at the molecular level may be the basis that SNPs affect the functional and structural connectivity of the brain, as well as the volume and density of gray matter and white matter.

The effects produced by SNPs represent information at the cellular, molecular, and other microscopic levels, whereas MRI can provide a macroscopic view of brain phenotypes. The approach of combining micro-level and macro-level information for research exemplifies the trans-scale research strategy. Methods that combine genetic information with imaging information fall under the umbrella of multi-omics technologies. On the one hand, the trans-scale and multi-omics analysis strategy that combines genomics, connectomics, and radiomics is instrumental to visualize the link between functional genetic variants associated with schizophrenia and the imaging phenotype. On the other hand, the trans-scale and multi-omics analysis strategy linking gene variation with brain structure and function have further contributed to the advanced studies of molecular biological mechanisms behind brain phenotypes and clinical manifestations of schizophrenia. The goal of trans-scale neuroscience of psychiatric illnesses is to deepen insights of the relationship between alterations at different scales (van den Heuvel et al., 2019). Therefore, an updated overview of trans-scale properties of schizophrenia based on multi-omics research strategies is needed.

The effect of risk genes on structural connectivity and FC during executive tasks was found in a systematic review summarized by Gurung and Prata in 2015 (Gurung and Prata, 2015). In this review, we have carefully categorized the types of brain phenotype alterations and the location of SNPs separately. For alterations in brain phenotype, we address both structural and functional aspects. Based on the location of the SNPs, we have divided them into non-coding regions and coding regions to describe, respectively. The purpose of this article is to summarize the studies on the relationship between specific SNPs loci in schizophrenia-related genes and cerebral alterations based on trans-scale and multi-omics strategies in the past decade, as well as demonstrate the advantages of trans-scale and multi-omics research strategy through the relationship between SNPs and alterations in brain phenotypes. The candidate genes are listed as follows, and the specific SNPs loci and their possible effects on trans-scale properties of schizophrenia will be discussed in detail (zinc finger protein 804A [*ZNF804A*], calcium voltage-gated channel subunit alpha 1C [*CACNA1C*], neurogranin [*NRGN*], cholinergic receptor, muscarinic 3 [*CHRM3*], oligodendrocyte lineage transcription factor 2 [*OLTG2*], d-amino acid oxidase activator [*DAOA*], d-amino acid oxidase [*DAAO*], Disrupted in Schizophrenia Gene 1 [*DISC1*], nitric oxide synthase 1 [*NOS1*], *KIAA0319, N*-Methyl d-Aspartate 1 [*GRIN1*], Glutamate receptor 2 [*GRIA2*], microRNA 137 [*MIR137*], metabotropic glutamate receptor 3 [*GRM3*], contactin-associated protein-like 2 [*CNTNAP2*], Neuregulin1 [*NRG1*], glutamate receptor delta 1 [*GRID1*], and cyclin M2 gene [*CNNM2*]) (Table 1).

**Table 1:** MRI studies investigating the impact of SNPs on brain.

**SNP ID**	**Gene**	**Location**	**Patients/controls**	**Findings**	**References**
Functional connectivity					
rs6800381	*CHRM3*	Non-coding region	161/150	FC between the left rectus and right thalamus (as quantitative traits)	Wang et al. (2016)
rs12807809	*NRGN*	Non-coding region	59/99	FC between the hippocampus and bilateral middle cingulate gyri and left anterior cingulate gyrus (TT < CC/CT)	Zhang et al. (2019)
rs11146020	*GRIN1*	Non-coding region	55/0	Causality connections between the left and right dorsolateral prefrontal cortex	Cai et al. (2020b)
rs2038136, rs2038137;	*KIAA0319*	Non-coding region	28/27	Resting-state network in language-related regions (no affection)	Jamadar et al. (2013)
rs1344706	*ZNF804A*	Coding region	52/128	Degree centrality in the precuneus (AA > CC/CA)	Chen et al. (2018)
rs1344706	*ZNF804A*		92/99	FC between the left hippocampus and right DLPFC (AA < CC/CA)	Zhang et al. (2018)
rs1344706	*ZNF804A*		78/153 (working memory)	FC of the right DLPFC and left hippocampal formation (contrast CC > CA > AA)	Rasetti et al. (2011)
rs1059004	*OLIG2*	Coding region	55/53	FC between left olfactory cortex, left parahippocampal gyrus, left middle temporal pole, bilateral hippocampus, and bilateral amygdala (AA/CA < CC)	Cai et al. (2020a)
rs1059004	*OLIG2*		49/47	Nodal efficiency in the right precuneus and left middle temporal pole (CA < CC)	Lv et al. (2020)
rs2391191	*DAOA*	Coding region	11/9	Connectivity density and larger global efficiency (AG > AA)	Liu et al. (2014)
rs1006737	*CACNA1C*	Coding region	54/80 (verbal fluency task)	FC between the left precentral gyrus/inferior frontal gyrus and superior temporal gyrus (AA/AG < GG)	Tecelao et al. (2019)
rs3782206	*NOS1*	Coding region	78/0 (Stroop); 76/0	FC between the right IFG and bilateral DLPFC in the Stroop task and the resting state (TT/CT < CC)	Zhang et al. (2015)
rs821617	*DISC1*	Coding region	46/24	FC between the right precuneus and inferior frontal gyrus (AA < AG/GG)	Gong et al. (2014)
rs3918346	*DAAO*	Coding region	40/48 (verbal fluency task)	FC between the left precuneus and a distributed network (TT/CT < CC) FC between the right posterior cingulate and right precuneus and left insula (TT/CT > CC)	Papagni et al. (2011)
rs4504469	*KIAA0319*	Coding region	28/27	Resting-state network in language-related regions (no affection)	Jamadar et al. (2013)
rs3813296	*GRIA2*	Coding region	55/0	Descending pathway from the prefrontal lobe to the striatum (GT < TT)	Cai et al. (2020b)
rs11146020	*GRIN1*	Non-coding region	55/0	Interaction effect: ascending pathway from the bilateral pallidum to the right caudate and the bilateral dLPFC	Cai et al. (2020b)
rs3813296	*GRIA2*	Coding region			
Structural connectivity					
rs35753505	*NRG1*	Non-coding region	36/31	FA in the anterior cingulum (TT/TC < CC)	Wang et al. (2009)
rs7808623	*GRM3*	Coding region	74/87	FA in the anterior thalamic radiation and corticospinal tract, as well as a series of tracts connecting the frontal cortex to the cerebellum (GG > TG > TT)	Mounce et al. (2014)
rs1625579	*MIR137*	Coding region	83/63	FA in both right orbitofrontal region and left striatum(TT < GT)	Kuswanto et al. (2015)
rs2710126	*CNTNAP2*	Coding region	44/81	FA in the uncinate fasciculus (AA < AG, AA < GG)	Clemm von Hohenberg et al. (2013)
rs1344706	*ZNF804A*	Coding region	100/69	FA, axial diffusivity, radial diffusivity, and mean diffusivity (no association)	Wei et al. (2013)
Brain structure					
rs12807809	*NRGN*	Non-coding region	91/65	Cortical thinning: frontal, parietal, and temporal cortices (TT) Thalamic shape abnormalities: regions related to pulvinar and medial dorsal nuclei (TT)	Thong et al. (2013)
			99/263	Gray matter volume in the left anterior cingulate cortex (TT < TC < CC)	Ohi et al. (2012)
rs3814614	*GRID1*	Non-coding region	62/54	Gray matter density in the right medial cerebellum and an area in the medial parietal cortex between the central and precuneal regions (in the cerebellar: CC < CT) (in the parietal: CC > CT)	Nenadic et al. (2012)
rs1344706	*ZNF804A*	Coding region	80/69	White matter density in the left prefrontal lobe and bilateral hippocampus (TT/GT > GG)	Wei et al. (2012)
rs7914558	*CNNM2*	Coding region	173/449	Gray matter volumes in the bilateral inferior frontal gyri (GG < GA/AA)	Ohi et al. (2013)
rs3813296	*GRIA2*	Coding region	55/0	White matter volume in the superior corona radiata (GT > TT)	Cai et al. (2020b)

*FC, functional connectivity; FA, fractional anisotropy; CHRM3, cholinergic receptor, muscarinic 3; CNTNAP2, contactin-associated protein-like 2; COMT, catechol-O-methyltransferase; DAAO, d-amino acid oxidase; DAOA, d-amino acid oxidase activator; DISC1, disrupted in schizophrenia gene 1; DLPFC, dorsolateral prefrontal cortex; FA, fractional anisotropy; FC, functional connectivity; IFG, inferior frontal gyrus; NOS1, nitric oxide synthase 1; NRGN, neurogranin; RS, resting state; ZNF804A, zinc finger protein 804A*.

## MRI and SNPs in Schizophrenia

### Brain Function and SNPs in Schizophrenia

#### Single-Nucleotide Polymorphisms of Non-Coding Region

In first-episode treatment-naive schizophrenia, FC network analysis has suggested significant effects of *CHRM3* rs6800381 on the abnormal thalamo-orbital frontal cortex connectivity (Wang et al., 2016). Compared with C allele carriers, *NRGN* gene rs12807809 TT homozygotes in patients with schizophrenia have significantly lower hippocampus-seeded FC values in bilateral middle cingulate gyri and left anterior cingulate gyrus, suggesting that rs12807809 may be involved in the pathophysiological process of abnormal Papez circuit function (Zhang et al., 2019). The effects of *GRIN1* rs11146020 are mainly reflected on the causality connections between the bilateral dorsolateral prefrontal cortex (DLPFC) (Cai et al., 2020b).

However, some SNP genotypes are not associated with changes of FC in patients with schizophrenia, demonstrating the uncertainty of genetic factors at the molecular and cellular levels. For example, left Broca superior/inferior parietal network and bilateral Wernicke-frontoparietal network are related to *KIAA0319* SNPs (rs2038136, rs2038137) only in controls, respectively, but not in schizophrenia (Jamadar et al., 2013).

#### Single-Nucleotide Polymorphisms of Coding Region

Chen et al. identified that rs1344706 within intron 2 of the *ZNF804A* gene played a role in degree centrality in the precuneus, an important hub of the whole-brain network, in patients with schizophrenia (Chen et al., 2018). The investigation on the relationship between rs1059004 polymorphism which locates in the 3′-untranslated region (3′UTR) intronic region of the *OLIG2* gene and the whole-brain FC in patients with first-episode schizophrenia reveals that the FC strength decreased both in patients with schizophrenia and healthy controls with risk A allele and there is at some level a positive relationship between FC strength and verbal fluency score in patients, suggesting that there are synergistic effects between rs1059004 polymorphism and brain connections (Cai et al., 2020a). Compared with C allele homozygote, patients with schizophrenia with risk A allele have significantly lower nodal efficiency in the right precuneus and left middle temporal pole (Lv et al., 2020). Using brain connectivity network properties, “AG” carriers of *DAOA* rs2391191 have higher connectivity density and larger global efficiency than “AA” carriers (Liu et al., 2014).

For the *CACNA1C* rs1006737, the risk allele carriers (AA/AG) show decreased connectivity between the left precentral gyrus/inferior frontal gyrus and superior temporal gyrus vs. non-risk allele homozygotes (GG) in schizophrenia, thus presenting abnormal verbal fluency (Tecelao et al., 2019). *ZNF804A* rs1344706 seems to play an important role in FC between the left hippocampus and right DLPFC, which may serve as the brain mechanism of rs1344706 in schizophrenia (Zhang et al., 2018). However, during a working memory task, seeded connectivity analysis of the homozygous control group of the risk allele (AA) demonstrate a disruption in right DLPFC–left hippocampal formation coupling when compared with the other genotype groups, but there is no effect of genotype in patients with schizophrenia (Rasetti et al., 2011). FC between the right inferior frontal gyrus and bilateral DLPFC is reduced in the risk allele carriers (the TT/TC group) of *NOS1* gene rs3782206 in both Stroop task and resting state, suggesting a relevance of rs3782206 to cognitive functions and neural mechanisms at the inferior frontal gyrus (Zhang et al., 2015). Significant association is also detected between the right precuneus inferior frontal gyrus functional connection and the *DISC1* rs821617 in patients with schizophrenia (Gong et al., 2014). For *DAAO* rs3918346 genotype, there are verbal fluency task-dependent changes of FC between the left precuneus and distributed networks including left and right precuneus, left putamen, right posterior cingulate gyrus, left caudate and right angular gyrus, and between the right posterior cingulate and right precuneus and left insula among patients with schizophrenia (Papagni et al., 2011). Similar to the effect of *KIAA0319* SNPs (rs2038136,rs2038137) genotype, the influence of SNP rs4504469 located at the exon of *KIAA0319* coding region on the left Broca upper/lower parietal network and bilateral Wernicke-frontalparietal network is reflected only in controls (Jamadar et al., 2013). In addition to investigating the influences of SNP rs11146020, which is located in the non-coding region of *GRIN1*, Cai et al. (2020b) found that SNP rs3813296 located in the intron region of *GRIA2* also has certain effects on the causality connections which located on the descending pathway from DLPFC to the striatum and thalamus in patients with schizophrenia. In the meantime, the interaction effects of rs11146020 and rs3813296 on causality connectivity are mainly located in the upstream pathway from the bilateral pallidum to the right caudate and the bilateral DLPFC, and negatively correlated with the Mayer–Salovey–Caruso emotional intelligence test, managing emotions score.

### Brain Structure and SNPs in Schizophrenia

#### Single-Nucleotide Polymorphisms of Non-Coding Region

As for structural connectivity and brain structure, MRI studies are helpful to explore biological clues about the genetic underpinnings of structural connectome deficits in schizophrenia (Voineskos, 2015). For the *NRG1* rs35753505 genotype, fractional anisotropy in the anterior cingulum of patients with schizophrenia with the T allele is significantly lower than that of patients with schizophrenia with CC genotype and healthy controls with T allele (Wang et al., 2009). On the basis of previous studies, Nenadic et al. (2012) found that the SNP rs3814614 located in the *GRID1* promoter region affected the gray matter density in the right medial cerebellum and a region of the medial parietal cortex. Moreover, the cerebellar cluster gray matter density of TT homozygous patients was the highest, CT heterozygote was the intermediate, and CC homozygote was the lowest, showing significant interaction effects of group × genotype (Nenadic et al., 2012). These findings contribute to our understanding of the mechanisms of the abnormal cortical–subcortical brain networks in schizophrenia with the involvement of the *NRGN*. Apart from the effect on FC, the *NRGN* rs12807809 genotype is also associated with the morphological and structural changes of the cerebral cortex. The frontal, parietal, and temporal cortices of patients with schizophrenia with TT genotype were extensively thinned, and there are also thalamic shape abnormalities in the regions involving pulvinar and medial dorsal nuclei (Thong et al., 2013). Furthermore, patients with schizophrenia carrying risk T allele have a smaller gray matter volume in the left anterior cingulate cortex, compared to non-risk C allele carriers (Ohi et al., 2012).

#### Single-Nucleotide Polymorphisms of Coding Region

The minor allele of rs7808623, located in the intronic region of *GRM3* gene, is associated with higher white matter integrity in the anterior thalamic radiation and the corticospinal tract, as well as a series of tracts connecting the frontal cortex to the cerebellum (Mounce et al., 2014). This study indirectly mirrors the importance of *GRM3* in maintaining white matter integrity. For *MIR137* rs1625579 genotype, patients with schizophrenia with risk T allele homozygous genotype decreased fractional anisotropy values in both right orbitofrontal region and left striatum compared to G allele/A allele carriers (Kuswanto et al., 2015). There are some correlations between *CNTNAP2*, also known as Neurexin 4 (*NRXN4*), rs2710126 genotype and fractional anisotropy in the uncinate fasciculus (Clemm von Hohenberg et al., 2013). Despite the lack of association between *ZNF804A* rs1344706 and white matter integrity in schizophrenia (Wei et al., 2013), T allele carriers present higher white matter density in the left prefrontal lobe and bilateral hippocampi (Wei et al., 2012). Compared with non-risk A allele carriers, patients with schizophrenia with G/G genotype of risk variant rs7914558 which is located in intron1 of the *CNNM2* have smaller gray matter volumes in the bilateral inferior frontal gyri, especially the orbital region (Ohi et al., 2013). Among the effects of *GRIA2* gene rs3813296 on white matter (Cai et al., 2020b), the most significant effect is located on the bilateral superior corona radiata fibers. Compared with the TT genotype, patients with GT genotype have a significantly larger volume of the superior corona radiata, which leads to the dispersion of the connection strength between the left DLPFC and the right caudate (Cai et al., 2020b). This is seemingly the factor that patients with GT genotype have a decrease in connection strength between the two areas. All the above research results provide a possible mechanism underlying the association between cerebral abnormalities and schizophrenia at the level of genetic polymorphisms.

## Discussion

**Figure 1:**
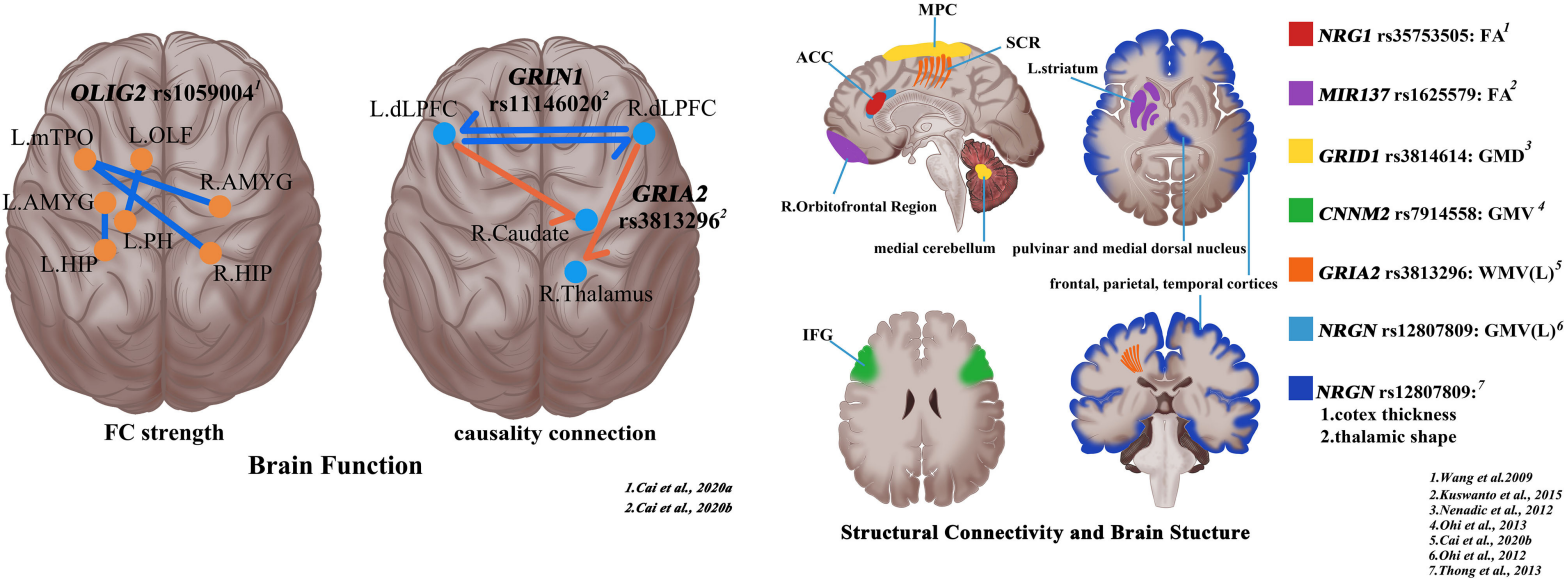
Shaping the trans-scale and multi-omics properties of schizophrenia *via* magnetic resonance imaging and single-nucleotide polymorphisms (SNPs). R, right; L, left; ACC, anterior cingulate cortex; MPC, medial parietal cortex; SCR, superior corona radiata; IFG, inferior frontal gyrus; FA, fractional anisotropy; GMD, gray matter density; GMV, Gray matter volume; WMV, white matter volume.

Based on the genetic variation data provided by GWAS research results, the current research takes the common SNPs in the whole genome as the objects to carry out association analysis at the overall level, and looks for the SNPs and susceptibility genes related to schizophrenia. The discovery of SNPs function increases the understanding of the association between functional genetic variation and imaging phenotypes related to schizophrenia from the gene level, and may also provide important clues about the anatomical heterogeneity of schizophrenia. Microscopic level alterations in gene function provide theoretical support for macroscopic level alterations in brain phenotype (van den Heuvel et al., 2019). The application of multi-omics method combining gene and MRI in schizophrenia intuitively reveals the possible pathogenesis related to genes from the perspective of brain structure and function changes (Figure 1). In particular, using genetic imaging strategies, especially based on the high-quality images presented by MRI techniques, to investigate the influence of genetic factors on brain phenotypes will help to study schizophrenia in a more integrated perspective. The key of the trans-scale and multi-omics research strategy is to synthesize the information of different scales and different disciplines, so as to provide the most comprehensive way to explain the nature of schizophrenia (Guan et al., 2021). A recent review systematically summarizes the application of multi-omics approaches in schizophrenia in terms of pathogenesis, disease typing, clinical grading, risk prediction, and precision interventions (Guan et al., 2021). Compared to the information provided by a single discipline, the combination of genetic data and imaging data can provide us with more comprehensive information. In other words, the trans-scale information provided by multi-omics methods can deepen the understanding of the pathophysiological mechanism of clinical symptoms of schizophrenia, and can provide new clew for the stratification of patients and high-risk groups and the development of more accurate risk and treatment response biomarkers. The analytical framework that combines clinical data from multi-view biclustering analysis with gene expression levels allows for the accurate identification of subtypes of schizophrenia (Yin et al., 2019). Protein interactome can be used to describe polygenic associations between antipsychotic drug targets and risk genes, and help to develop new targets for the treatment of negative symptoms and cognitive impairment in schizophrenia (Kauppi et al., 2018). Clinical transformation is one of the ultimate goals of all research. The heterogeneity of schizophrenia is indirectly reflected by the multiple imaging features exhibited by patients with schizophrenia. Different genotypes of patients also have different brain phenotypes, which show individualized characteristics in neuroimaging. This heterogeneity between different scales indirectly suggests that clinicians need to design more individualized and precise clinical decisions.

Most recently, functional striatal abnormalities have been developed as a new neuroimaging biomarker for the identification, prognosis, and subtyping of schizophrenia based on brain function (Li et al., 2020). Loci of striatal hyperactivity recapitulate the spatial distribution of dopaminergic function and the expression profiles of polygenic risk for schizophrenia. Furthermore, by applying a novel machine learning method, 413 genetic factors related to schizophrenia across 13 brain regions can be obviously identified (Huckins et al., 2019). The expression of schizophrenia-related genes is reflected in the whole neurodevelopmental process: some during specific stages of pregnancy, and others during adolescence or adulthood. Genetic influence on schizophrenia paves the way for the potential application of MRI in schizophrenia (Jiang et al., 2020). A network fusion-based approach has been applied to integrate three types of data, including genetic, epigenetic, and neuroimaging data, for the diagnosis and prediction of patients with schizophrenia (Su-Ping et al., 2016). For example, adolescents in a high-risk state can be screened by identified risk factors for schizophrenia, so as to predict and intervene at early stage in the future for adolescents who may suffer from schizophrenia. Future research will place more emphasis on integrated analysis of information across different dimensions supported by trans-scale and multi-omics technologies (van den Heuvel et al., 2019; Guan et al., 2021). This new trans-scale and multi-omics methods give us unprecedented power to understand the nature of schizophrenia.

But so far, this combination method has only played a hint and reference role in the pathogenesis of schizophrenia. Most of the studies involved in this article are hypothetical, only with the help of imaging methods to observe changes in brain structure and function in the presence of a specific SNPs. The researchers did not conduct animal experiments to confirm that the changes shown in the images were induced by the specific SNPs. This is a common problem in related research fields. At the same time, due to the complexity and genetic heterogeneity of schizophrenia, there are differences among regions, races, and populations. Therefore, experiments need to reduce the contingency and increase the universality of results. In addition, the sample size of most studies is relatively small, especially the studies on the effects of the relationship between genes and structure on the brain are based on healthy people. It is necessary to increase the sample size for the verification of the results. Furthermore, future research will place more emphasis on integrated analysis of information across different dimensions supported by trans-scale and multi-omics technologies. This is a great challenge to be faced in future research. It is also important to note that the use of antipsychotic drugs can lead to some alterations in the brain phenotype of patients with schizophrenia (Guo et al., 2019; Wang et al., 2019). Future studies need to be aware of whether or not patients with schizophrenia have been treated with antipsychotic medication when discussing changes in their brain phenotypes.

All in all, the new trans-scale and multi-omics methods give us unprecedented power to understand the nature of schizophrenia, which will promote the identification of biomarkers and risk prediction ability, provide help for the multigene strategy of heterogeneous dissection of schizophrenia, and further promote the implementation of individualized accurate diagnosis and treatment.

## Author Contributions

Y-HZ, HY, and L-BC conceptualized the manuscript. S-WZ, XX, and L-BC wrote the first draft of the manuscript. All authors provided feedback and revised the manuscript.

## Funding

This work was supported by the grant support of Fourth Military Medical University (2019CYJH) and the Project funded by the China Postdoctoral Science Foundation (2019TQ0130).

## Conflict of Interest

The authors declare that the research was conducted in the absence of any commercial or financial relationships that could be construed as a potential conflict of interest.

## Publisher's Note

All claims expressed in this article are solely those of the authors and do not necessarily represent those of their affiliated organizations, or those of the publisher, the editors and the reviewers. Any product that may be evaluated in this article, or claim that may be made by its manufacturer, is not guaranteed or endorsed by the publisher.
